# Supplementary material for: Mathematical Modelling Using Predictive Biomarkers for the Outcome of Canine Leishmaniasis upon Chemotherapy
Source: Microorganisms. 2020 May 15;8(5):745. doi: 10.3390/microorganisms8050745 (PMC7285289; doi:10.3390/microorganisms8050745)
Supplement: Supplementary file 1 [file microorganisms-08-00745-s001.pdf]

Supporting Figure 1

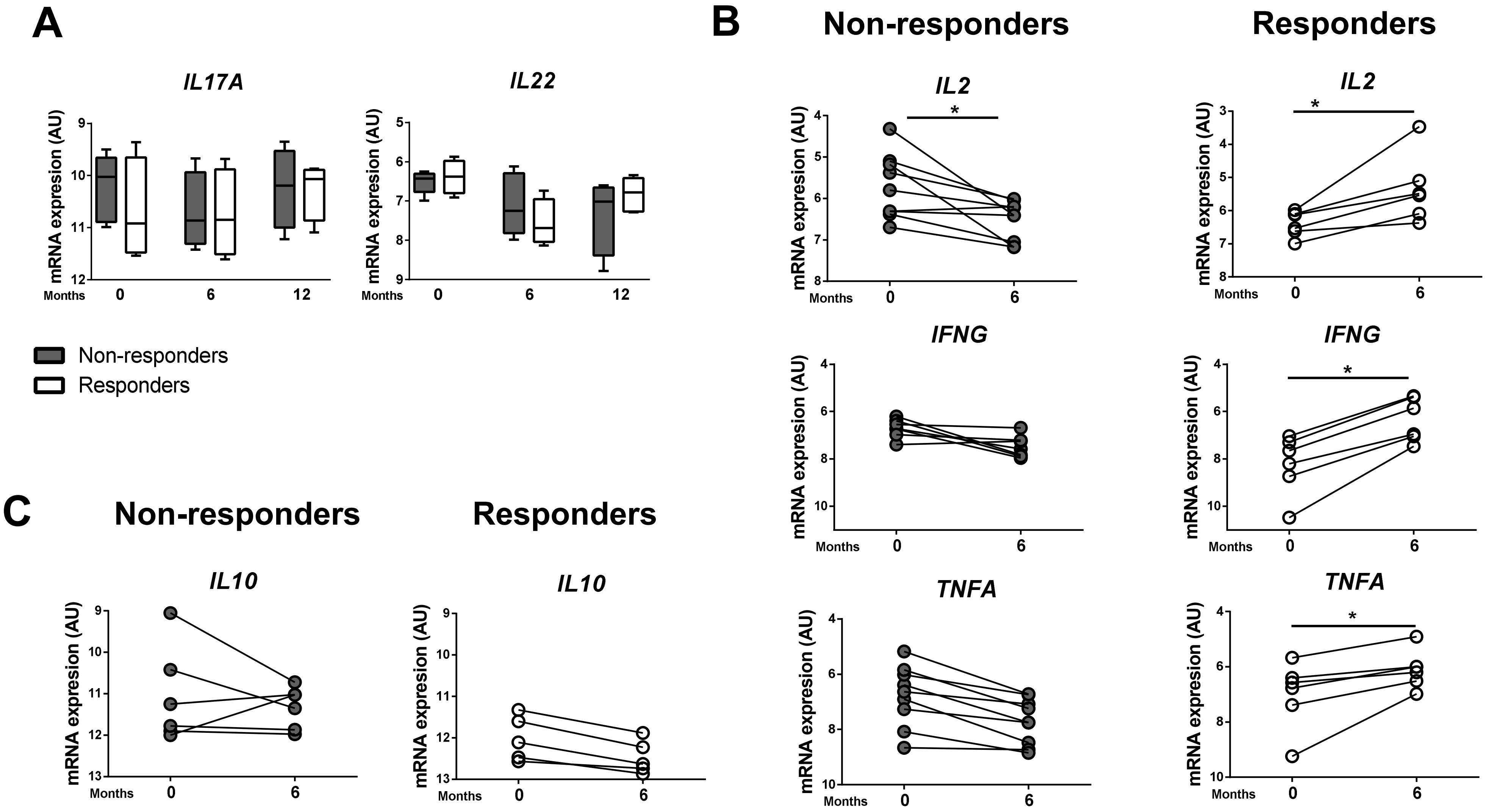

Supporting Figure 1. The success of anti-*Leishmania* chemotherapy is independent of a Th17 response. mRNA was isolated from spleen biopsies at T0, T6 and T12. Quantitative PCR was performed for *IL17*A and *IL22* (A). Individual plots for *IL2*, *IFNG* and *TNFA* mRNA expression at T0 and T6 (B). Individual plots for IL-10 mRNA expression at T0 and T6 (C). Data are shown as mean ± SD or in a box and whisker plot format, n=5-10 dogs/group. *p<0.05.

Supporting Figure 2

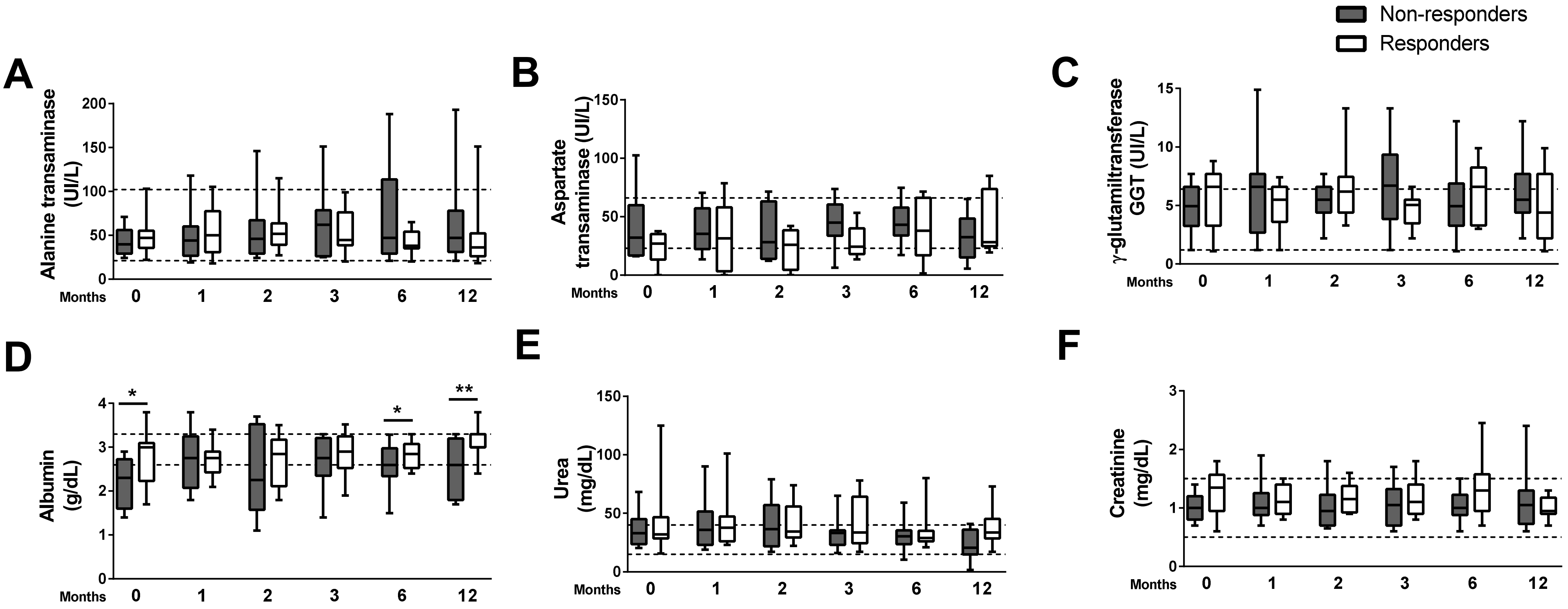

Supporting Figure 2. Hepatic and renal parameters in the serum and urine, respectively, of non-responder and responder dogs. The serum aspartate transaminase (A), alanine transaminase (B), gamaglutamiltranspeptidase (C) and albumin (D) as well as urine urea (E) and creatinine (F) values was quantified at T0, T1, T2, T3, T6 and T12. Data are shown as mean ± SD or in a box and whisker plot format, n=12-14 dogs/group. *$p<0.05$; **$p<0.01$.
